# Analecta

**Published:** 1842-06-11

**Authors:** 


### ANALECTA.

*Excision of a Large Tumour upon the Neck.* By R. D. Mussey, M. D., Professor of Surgery in the Medical College of Ohio.—In December, 1841, I was consulted by Mr. Jas. C. M'Dowell, æt. 34, of Mt. Carmel, Wabash county, Illinois, on account of a large tumour on the right side of the neck, and received from him the following account of it.

The tumour commenced eighteen years ago, in the form of a lump, below the tip of the ear, of the size of a hazelnut, which was painful, and which on that account was thought to be mumps. The pain subsided in a few days, but the swelling and hardness remained. From that time the progress of the tumour was slow, and almost always without pain, till within the last eighteen months, during which time he had experienced a great deal of pain in the ear and on the side of the face. In 1828, between five and six years after the first appearance of the disease, and when the tumour was about the size of a hen's egg, he came to Cincinnati and took advice from the Professor of Surgery in the Medical College of Ohio, who declined operating, saying, "that the carotid artery must first be tied, or the extraction of the tumour would prove fatal in a few minutes; and besides, the right side of the face would be palsied by the division of an important nerve," &c.

The tumour presented, at the time above mentioned, viz., December, 1841, the following appearances: It was nearly hemispherical in form, with some tuberosities, extending from the lower part of the concha of the ear, which it crowded a little upward, to within an inch and three quarters of the clavicle, and antero-posteriorly from the anterior border of the cervical portion of the trapezius, to within two inches of the median line upon the chin, covering part of the larynx and trachea, and a large portion of the lower jaw. A line stretched from the anterior to the posterior edge of the base of the tumour, over its apex or pole, measured ten inches; and its circumference at the base was seventeen inches. The sterno-cleido-mastoid muscle was put in a state of tension upon the back part of the tumour, and seemed adherent to it. This large mass possessed a good degree of solidity, had no uncommon sensibility to the touch, could be made to glide slightly in the antero-posterior direction, showing that it did not involve the deep and large vessels, and most important nerves of the neck: the integument covering it was healthy looking.

I decided upon the practicability of its removal, and put the patient upon a farinaceous diet, with water only for his drink; and on the 11th of January, 1842, in the presence of several professional gentlemen, and a few friends of the patient, proceeded to the operation. The integuments and platysma were divided by a vertical and horizontal incision crossing each other at right angles upon the pole of the tumour; the flaps were carefully raised, and the mass slowly disengaged from the condensed cellular bands which shot from the neighbouring parts, and from the mastoid muscle, a portion of the attenuated edge of which was removed. Some difficulty was found and a good deal of pain produced in detaching it from the infra auricular and infra maxillary tissues, but no important bloodvessel was wounded or muscle mutilated, except the mastoid; nor nerve injured, except a descending branch of the facial, by which a slight displacement was given to the integuments of the chin. The angle of the mouth kept its natural position. There was less than a pint of blood lost, and the patient, though somewhat faint for a short time during the operation, causing a little delay, had so far rallied as to be comfortable during the application of the dressings, and after he was carried to his bed. The following night he was restless and had considerable pain with irritative fever, which were soothed by an anodyne dose with spiritus mindereri.

After the first night Mr. M'Dowell was comfortable—the wound healed kindly, and in four weeks he left the city to visit his friends. Within the last few days we learn by a gentleman directly from his residence, that he is in sound health and good spirits.

For the purpose of safely extracting large tumours from the neck, it can rarely be necessary to ligate the carotid artery as a preparatory step. By carrying the dissection close to the morbid growth but little risk is incurred, unless in the fungoid growths, which sometimes completely encircle large vessels and important nerves; and with these there is but little encouragement for an operation.

The slow progress of the tumour, together with its solidity and freedom from irritation, served to mark it as a morbid structure of mild character; and yet from the frequent and strong pains induced by mechanical tension of sentient nerves in its neighbourhood during the last year and a half, it might ultimately have kindled up an action, the result of which would be obstinate or incurable ulceration; but, as it is, the operation will almost certainly be followed by entire exemption from the disease.

The superficial portion of the parotid gland was not to be observed distinct from the tumour; indeed there was no obvious trace of any part of it remaining. Like most tumours of slow growth, occupying the site of the parotid, it commenced, in all probability, in a lymphatic gland, and by pressure during its progressive and protracted enlargement, it had caused an entire absorption of so much of the parotid as came in its way.—*Western Lancet.*

---

*M. Velpeau's Mode of administering Cubebs and Copaiva in Gonorrhœa.* [Extract from a Lecture at La Charité.]—First I would remark, that any of you who have followed my practice in this hospital must be convinced of the error of those who assert that cubebs is more liable to irritate than copaiva. Eighteen out of twenty patients will bear the remedy well. One patient labouring under an old gonorrhœa had completely lost his appetite, and was much enfeebled; after he had taken the cubebs for some time his health was visibly improved. On the other hand, several patients were compelled to renounce the use of copaiva, when administered by itself.

Cubebs, then, is a medicine more easily supported, but it is less efficacious. The following is the mode in which I am accustomed to administer it:—I commence by giving a drachm thrice a day, at morning, noon, and night. If the stomach bear it well, I gradually increase the dose to six drachms daily. It must be given in powder, and you should take care to procure it fresh, for I am convinced that cubebs loses much of its efficacy by being kept for any length of time. Many of you have seen the discharge arrested on the second or third day by cubebs; in some cases even within twenty-four hours. It is, therefore, impossible to doubt its efficacy. But you should not continue it beyond the fourth day; if it produce no effect by that time, it may be laid aside as useless.

Copaiva is the next remedy which we have to notice. It is, as you all know, excessively disagreeable, and gives rise to nausea, vomiting, &c.; hence many attempts have been made to mask its taste. Chopart mixed it with alcohol and other substances; but many patients cannot bear even Chopart's mixture. It has been mixed with sugar, but the nauseous taste still predominates; besides, the efficacy of the remedy is diminished in proportion to the saccharine qualities of the substances mixed with it.

Being desirous of avoiding the effects of copaiva on the stomach, I made several trials of this remedy in the form of enema. From one to four drachms of copaiva were mixed with four ounces of gum emulsion, containing some camphor and opium. I began with a drachm of the copaiva, and increased the dose by a drachm every day; the enema was thrown up thrice daily. There was a great obstacle, however, to this mode of treatment. Most of the patients were unable to keep the enema; but those who did were cured in three or four days. From these facts, and others observed by different surgeons since, I am inclined to think that this mode of giving the balsam, when the patient can retain it, is as efficacious as any other.

The best method, Gentlemen, is to unite the two substances together. The following is the formula for the mixture which I have been in the habit of employing for several years:—Two drachms of copaiva, four to six drachms of cubebs, and two grains of opium, are made up into a paste with magnesia; this is divided into six parts, and taken in two days. Three doses are generally sufficient to effect the cure. In some cases the discharge is arrested after the second day; but you must not suspend the remedy as soon as this occurs, for the discharge would return in greater abundance. You must continue your treatment for some time. After the first dose, allow the patient to rest for a day; on the fourth day you give another dose, and on the seventh a third. Some writers pretend that the use of copaiva and cubebs is apt to induce metastasis of the inflammation to the joints, &c. I do not think that this is the case; a mere coincidence has been confounded with an effect. It is true that arthritis frequently occurs in persons labouring under gonorrhœa, and the discharge may be a predisposing source of this affection; but we are not to conclude from this that gonorrhœal arthritis arises from the use of cubebs and copaiva. The occurrence of a rash, somewhat similar to measles, is more evidently connected with the administration of these remedies; but the effect is of so little consequence that it scarcely deserves notice.

It is impossible to deny that the remedies just mentioned exercise a certain influence on the urinary organs. The urine is strongly impregnated with their odour; some writers pretend that they act as revulsives; but I cannot admit this, for their anti-gonorrhœal effect is greater in proportion to their want of action on the digestive tube. In a word, the best constitutional treatment of gonorrhœa consists in the administration of copaiva and cubebs united together.—*Provin. Med. Jour.* April 16, 1842.

---

*Urethroplasty.*—At the meeting of the Academy of Medicine, Paris, March 28th, 1842, M. Segalas communicated the details of the following case:—A patient, when six years of age, tied a piece of string round the penis; the results were gangrene of the organ, and the formation of a fistulous opening, which gave passage to the urine and semen. The orifice in the urethra was an inch in length, and the portion of canal anterior to it was very considerably contracted.

M. Segalas placed a catheter permanently in the bladder, and divided the prepuce, to make the integuments more lax. When the wound was healed, he passed a catheter again, to guide the bistoury, with which he opened freely the membranous part of the urethra; he then withdrew the catheter, introduced a gum-elastic bougie, and converted the latter into a syphon, with an œsophagus bougie. A few days afterwards he made two semilunar incisions, before and behind the fistulous opening, dissected off the skin, and then brought the edges of the wound together with six points of twisted suture. A bougie had been passed into the wound of the membranous part of the urethra, and brought out, in front, through the meatus urinarius: the penis was now enveloped in lint dipped in cold water, and the syphon put into action. The edges of the wound quickly healed at every point, except near the left side, where the sutures were too far asunder; this, however, soon contracted, and an excessively small orifice only remained. At the end of two months the wound in the perineum was allowed to heal up, but a few drops of urine now escaped from the pin-hole already alluded to. This was cauterised several times without effect; its edges were therefore resected, and brought together by a single suture. The fistulous opening was definitely cured by the last operation.—*Ibid.* April 23, 1842.

---

*Pathology of Sudden Death.* By R. H. Semple, Surgeon.

Case I.—*Sudden Death from Disease of the Heart.*—John Charter, ætat. 58, admitted as a patient into the Islington Infirmary, Sept. 19, 1835; he had been employed for several years as an ostler, and was a man of intemperate habits. He had generally enjoyed good health, till a few weeks before his admission, when he was attacked with an affection of the chest, for which he received no medical advice. The symptoms on his admission were as follows:—He complains of pain and tightness in his chest with difficulty of breathing; he lies indifferently on either side; is very restless in bed, and is obliged to have his head supported in consequence of the urgent dyspnœa; tongue clean; pulse moderate, but hard and wiry; abdomen swelled; legs anasarcous; face pale; urine rather scanty; scrotum dropsical; bowels not much confined; little appetite; some thirst. The osculta-tion of the chest proved that the heart and lungs were diseased; the impulse of the former being very strong, but without any morbid sound; and the latter giving evidence of inflammation of the air-tubes by sonorous and sibilant rhonchi heard on both sides of the chest. This man was ordered to lie in bed with his head elevated, to drink toast and water, or acidulated drinks, and to use a moderate diet. He was also ordered a blister to be applied to the chest, and the following medicines:—℞. Jalap powder, ʒj.; Bitartrate of potash, ʒij. ft. The powder to be taken three times a-day. ℞. Solution of iodide of potassium, m. xx.; Mint water, ℥j. ft. The draught to be taken every four hours.

These medicines caused him to pass a great quantity of watery evacuations, and afforded him some relief; they were continued, with some alteration of the quantities, until Oct. 2d, when the abdomen was so enormously distended with fluid, that paracentesis was deemed advisable, and this operation was accordingly performed. Three gallons of straw-colored serum were removed, and he expressed himself very much relieved. His health began gradually to improve, he rose from his bed, and was able to walk about the ward, and he appeared rapidly to be approaching to his ordinary state of health. But on the 23d of December, twelve weeks after tapping, and more than three months after his admission, he expired suddenly while walking to the water closet.

On examining the body, the heart was found much increased in size; the left ventricle was hypertrophied and dilated, but there was no disease of the valves. The lungs were congested, and the bronchial tubes thickened and inflamed, and filled with frothy mucous. The brain was not examined.

Case II.—*Sudden Death from Disease of the Heart.*—James Miles, ætat. 55, admitted as a patient into the Islington Infirmary, January 15, 1840. The impulse of the heart was very strong, and heard over a large space; the lips and face were livid; the legs anasarcous. He was ordered the following:—℞. Tincture of digitalis, m. x.; Infusion of senna, ʒx.; Epsom salts, ʒij. The draught to be taken every four hours. ℞. Bitartrate of potassa, ʒij.; Nitrate of potassa, gr. x. The powder to be taken twice a-day.

This man died suddenly four days after his admission.

*Post-Mortem Examination.*

*Chest.*—The heart was enlarged to about twice its natural size; the parietes of both ventricles were hypertrophied, but their cavities were of the natural size. The pulmonic semilunar, the mitral, and the tricuspid valves were healthy, but the aortic semilunar valves were rigid and cartilaginous. The aorta was much dilated to the distance of about four inches from its origin, and the pouch so formed presented internally a large quantity of calcareous particles, arranged in plates and scattered in granules. This osseous deposition was traced at intervals along the whole course of the thoracic and abdominal aorta as far as its bifurcation. The left lung was healthy, and the bronchial tubes did not present any marked morbid appearance. The right lung was adherent to the parietes of the chest by strong fibrous bands; the lower part of this lung was hepatised and very friable, the middle portion was engorged, the upper part healthy; in other words, the inferior and middle portions of this lung exhibited the first and second stages of pneumonia. The existence of pneumonia as a consequence or an accompaniment of diseased cardiac valves, has been pointed out by many authors, and I have seen several cases of the kind in my own experience.

*Abdomen.*—The liver was large, and presented the nutmeg appearance; the other viscera of the abdomen were healthy. The brain was not examined.

In this case there were preparations evidently made for an aneurism of the aorta, had not death put an end to the process. The existence of osseous matter in the lining membrane of the arteries, although common enough in advanced age, is by no means frequently found in persons so little advanced in years as the subject of the present account.

In the two cases just detailed, death must be attributed to a spasmodic affection of the heart causing a sudden cessation of its action; a result which was anticipated from the great increase in size of that organ. But in the next case, which is a very curious one, although the heart and lungs were, no doubt, originally in fault, the immediate cause of death was a fatal and profuse hæmorrhage from the mucous surfaces.

Case III. *Sudden Death from Profuse Hæmorrhage.*—John Carter, ætat. 78, was taking down some article from a shelf when he was suddenly seized with a profuse vomiting of blood, and died in a few minutes. I attended immediately afterwards, and found a large quantity of blood on the floor, and about a quart in a basin; it appeared of a florid colour. The mouth, nostrils, and face of the man were covered with blood.

*Post-Mortem Examination twenty-four hours after Death.*

On removing the sternum the right lung appeared very large, and extended over to the left side; it was of a dark colour, and did not collapse on the removal of the sternum. On its anterior and inferior surface a dark, blackish patch appeared, about the size of a hen's egg: on cutting into which, it presented a dark, bloody, congested appearance, not, however, bounded by any definite border, and resembling the disease called by Laennec apoplexy of the lungs. Other similar congestions were seen in other parts of the right lung. The left lung was extremely small, collapsed, and impermeable to air, except at a very few points near the edge; there were strong adhesions on this side between the costal and pulmonary portions of the pleura, and the substance of the lung was soft and friable. On cutting into this lung, the edges were found permeable to air; the lower part was hard and fleshy, while the upper and middle portions appeared broken down and ulcerated, and the arteries were found crossing the cavities in the form of strong, hard cords. No phthisical tubercles, however, were seen in any part. At the point where the left pulmonary artery entered the lung, the passage was found to be impervious; and on further examination it was found to be closed up by the growth of a fleshy, dense substance around its walls. The heart appeared externally to be somewhat enlarged; the two auricles were found healthy, as was also the right ventricle; but the left ventricle was dilated and hypertrophied, the dilatation being slight, but the hypertrophy considerable; the ceptum ventriculorum very much thickened. The aortic semilunar valves were found hardened by a copious deposition of calcareous matter; there seemed, however, sufficient room for the passage of the blood. The aorta was found to be degenerated; patches of albuminous matter were secreted along its course, with atheromatous deposits situated below the lining membrane. The trachea was examined and found healthy; but on accidentally opening the œsophagus, blood was seen to issue from the wound, which circumstance led me to examine the stomach very carefully. This organ was large, and constricted in the middle. On opening it, it was found filled with an enormous quantity of dark, grumous blood, mixed up with mucous and half-digested food. On cleaning the stomach a layer of gelatinous bloody matter was found lying on its mucous membrane, and was removed with some tle difficulty. The mucous membrane was found in the stomach to be generally red and injected. No vessel, however could be found ruptured, though the cœliac axis and its arteries were found degenerated in the same manner as the aorta mentioned above. I considered this case as one of hæmatemesis; but Dr. Copland, who saw it reported in the "Medical Gazette," and has noticed it in his Dictionary, article Hæmorrhage, thinks, with more probability, that it was really one of hæmorrhage from the trachea and bronchial tubes, some of the blood making its way into the stomach and causing vomiting, and thus leading to an appearance of hæmatemesis. This explanation certainly accounts for a fact which perplexed me exceedingly, for while the blood on the floor of the room was quite florid, that in the stomach was darkened by the gastric juice.

In the three next cases which I am about to detail, the disease of the heart, which was the immediate cause of death, was complicated with congestive or inflammatory disease of the brain. This connection of cardiac with cerebral disease is very property insisted upon by several modern writers; and I may mention that in my own practice I have found disease of the heart accompanied by sanguineous apoplexy, meningitis, and ramollissement.

[Case IV. of sudden death from disease of the heart and meningitis scarcely belongs to the series; it should have been omitted.]

Case V.—*Sudden Death from Disease of the Heart, attended with Meningitis.*—John Grant, ætat. 49, a navigator, a strong, muscular, and well-formed man, of intemperate habits, living in Islington, had obtained a letter for the Islington Dispensary on account of cough and shortness of breath. He was not seen by the physician, either from some misunderstanding, or because he did not suppose, from the representations of the relatives, that it was a case of sufficient importance to require visiting. Indeed, it does not appear that his wife herself thought her husband in any immediate danger, for on the very morning of his death he had expressed himself better. While his wife was gone to the dispensary to request the physician to visit her husband, he was sitting up in bed, and asked for some beer, which was given to him by a woman in the room, immediately after drinking which he fell back in the bed and expired. I was sent for and came immediately, but he was dead before I arrived. A coroner's inquest sat upon the body, and returned a verdict of "natural death." A post-mortem examination had been previously made by order of the coroner, when the following appearances was observed:—

*Head.*—The scalp and skull presented no peculiar appearance. The vessels of the dura mater were injected. The arachnoid membrane was highly inflamed and thickened, and a considerable quantity of thin serous fluid was found between its layers. The pia mater was healthy. The substance of the brain was firm, healthy, and of its natural colour, and although cut in every direction, exhibited no signs either of abscess, congestion, or sanguineous effusion.

*Chest.*—The lungs were very much increased in size, and in a high state of congestion. The bronchial tubes exhibited the marks of intense and long-standing inflammation ; they were much thickened, and the lining membrane was of a deep-red colour, marked with red longitudinal striæ, and filled with a large quantity of frothy and muco-purulent matter. The heart was very much enlarged, being about twice its natural size, and very fat. The right ventricle contained some fibrinous clots ; the left ventricle some half-coagulated blood. The cavity of each ventricle, especially the left, was enormously distended ; the walls were of about the usual thickness, and the carneæ columnæ were of ordinary size : the case, therefore, was one of dilatation of the heart, with hypertrophy. Had the parietes of the ventricles been thinner than in the natural state the disease would have been simple dilatation, which is but rarely seen. The valves were healthy, and appeared to offer no obstruction to the current of the blood.

*Abdomen.*—The liver was congested, and of a dark colour. The stomach was healthy, and nearly empty. All the other viscera were quite healthy.

Case VI.—*Sudden Death from Disease of the Heart, attended with Sanguineous Effusion into the Brain.*—Jane Harwood, ætat. 68, an inmate of the Islington workhouse, had long suffered from difficulty of breathing and palpitations of the heart. The action of the heart was accelerated, its impulse very strong, and heard over a greater space than is natural; pulse very feeble and quick. She had been several times attacked with violent fits of dyspnœa and faintness, which had been relieved by diffusible stimulants, and mustard-poultices to the calves of the legs. She had also taken small doses of tincture of digitalis, tincture of opium, and gentle purgatives, with considerable benefit. As I had long anticipated sudden dissolution in this case, she was ordered to remain in the infirmary, to be kept perfectly quiet, and to have a light nutritious diet. But having been for some time previously in a comparatively good state of health, she was suddenly seized, on the evening of the 31st of May, 1841, with violent pain in the left side, with difficulty of breathing. When I visited her, very shortly after the attack, I found her *in articulo mortis*, with very rapid and feeble pulse, cold extremities, convulsive movements of the mouth, pale and bloodless lips, eyes fixed and half closed. Mustard poultices were immediately applied to the region of the heart and the calves of the legs, and hot bricks to the soles of the feet. The following medicine was prescribed :—

R. Compound tincture of lavender, m. x.
Mint water, ℥i.

but before it could be sent she expired.

*Post-Mortem Examination Thirty-six hours after Death.*

*Head.*—The scalp, skull, aud membranes of the brain were healthy. But on examining the left ventricle that cavity was found filled with an enormous coagulum of blood, which not only filled that ventricle, but had passed through the third ventricle, and occupied a great part of the right ventricle.

*Chest.*—There were some slight old adhesions in both lungs, and there was a considerable quantity of thin serous fluid effused into the cavities of the pleuræ on both sides. The lungs were voluminous, crepitant throughout, and emphysematous at their edges and on their surfaces. The bronchial tubes were of a dark-red colour on their internal surface, which was marked by longitudinal purple striæ, and contained a small quantity of frothy mucus. The pericardium contained a small quantity of thin serous fluid. The heart externally appeared about one-half larger than its natural size; on being opened it was found that the cavity of the left ventricle was somewhat diminished in diameter, but not to a great extent, while the walls were enormously thickened, and the carneæ columnæ of great size. The aorta and pulmonary artery were quite free from disease, and their valves were healthy.

*Abdomen.*—The liver, which was of its natural size, presented a veined and granular appearance, both externally and internally, resembling what is called the nutmeg liver. The kidneys were below the natural size; externally they presented several vesicles of about the size of a bean, and containing serum; internally the glandular tissue was absorbed, and its place in great measure supplied by fatty and fibrous matter.

In the three following cases of sudden death no disease of the heart could be detected.

Case VII.—*Sudden Death from Cerebral Congestion.*—Sarah Holt, ætat. 17, an inmate of the Islington workhouse, had been in the infirmary for some time on account of suppression of the menses. She was a stout, healthy-looking girl, of a florid complexion. She took the medicines usually prescribed for amenorrhœa, and became much better, but still the menstrual discharge was not restored. She left the infirmary, apparently cured, on Monday, March 1, 1842. On Tuesday she pursued her usual avocations, and about four o'clock in the afternoon engaged in a fight with another girl. After eating her supper she went to bed at the usual hour, and while in bed she complained of pain and uneasiness to the persons in the same ward; but her complaints were not of such a character as to induce her to ask for medical assistance. The next morning she was found dead in her bed, with her face downwards. A coroner's inquest was held upon the body, and as there were no circumstances of suspicion attaching to the case, a verdict of "natural death" was returned. After the inquest a post-mortem examination was made, when the following appearances were observed:—

*Head.*—The vessels of the scalp were turgid with blood; and on removing the skull the brain was found to be in a high state of congestion, the veins and sinuses being greatly distended with fluid blood. Beyond this general congestion of its vessels the brain presented no morbid appearance, and no extravasation could be detected in any part.

*Chest.*—The heart was perfectly healthy, as were likewise the lungs, with the exception of some old adhesions between the two surfaces of the pleura.

*Abdomen.*—The stomach, intestines, and liver were all healthy. The bladder was empty. The uterus, which might have been supposed to exhibit some morbid appearance, was small, of healthy structure, and its cavity empty.

Case VIII—Is one of sudden death from cerebral congestion.

Case IX.—*Sudden Death from Syncope.*—Mrs. R., aged 43, a lady of great talents and accomplishments, residing in Islington, had been under the care of Dr. Babington for some weeks, in consequence of a pleuritic effusion into the right side of the chest. She was not considered in any danger, and her husband and son had both gone to town as usual, leaving her at home with the servant. Between three and four in the afternoon of Friday, March 4, she was in bed, and had occasion to use the night-pan, and soon after passing a motion she became suddenly very ill and faint; and though I and another medical gentleman were sent for and attended immediately, she expired before our arrival.

The next day, at five o'clock, Dr. Babington made the post-mortem examination, at which I was present. I then learned that Dr. B. had treated the case as one of effusion into the right side of the chest, for which he had prescribed a blister, with small doses of blue-pill, and tincture of opium, in combination with tonics. He had stated to the lady's husband that the disease was curable. The post-mortem examination will prove that Dr. B.'s prognosis and diagnosis were, at the time he made them, perfectly correct, although the former was falsified by some mysterious agency, the operation of which no human science could foresee or prevent.

*Chest.*—On inserting a trocar between the ribs on the right side, a straw-coloured fluid was poured out. Nearly two quarts of this fluid were found between the costal and pulmonary portions of the pleura on this side, the pleural surfaces being thickened and connected together by numerous delicate filamentous bands which traversed the fluid. The lung of this side was very much compressed, and the two lower lobes nearly obliterated. The left lung was quite healthy. The heart was pushed very far to the left side. This organ was flabby, the walls of about the ordinary thickness, the cavities slightly dilated, the valves all healthy. The blood in the heart, as well as in the other parts, was fluid. It is remarkable that there was no indication externally to denote effusion into the chest: both sides appeared to be of equal size, and no prominence was observed in the intercostal spaces. On percussion, however, the right side was quite dull, the left gave healthy sound.

*Abdomen.*—The liver was excessively enlarged, extending over to the left side, and downwards to the illiac region: its structure, however, only presented the appearance of great congestion. The spleen was congested and friable. The inner membrane of the stomach presented a dark red color and corrugated appearance, the results either of congestion or chronic inflammation. The kidneys, uterus, and intestines, were quite healthy.

The brain was not examined.

The cause of death in this case is involved in considerable obscurity; the fatal result cannot be attributed to the effusion into the chest, because empyema is by no means an incurable disease, or if fatal, it is never rapidly so; and as the other lung and the heart were healthy, the risk of sudden dissolution could not be reasonably anticipated. Dr. Babington attributes the death of the patient (and for want of any better explanation, I am induced to coincide in his opinion) to the fact that she became exhausted from the evacuation of the bowels, and sunk into a fatal syncope. Considering the large quantity of fluid contained in the right side of the chest, and pressing upon the heart, and the enormously enlarged liver also pressing npwards upon that organ, together with the enfeebled state of the patient, it is probable that the action of the heart might have become suddenly arrested by the slight excitement referred to, and a fatal termination induced.—*London Lancet, April* 16, 1842.

